# The balance of reproducibility, sensitivity, and specificity of lists of differentially expressed genes in microarray studies

**DOI:** 10.1186/1471-2105-9-S9-S10

**Published:** 2008-08-12

**Authors:** Leming Shi, Wendell D Jones, Roderick V Jensen, Stephen C Harris, Roger G Perkins, Federico M Goodsaid, Lei Guo, Lisa J Croner, Cecilie Boysen, Hong Fang, Feng Qian, Shashi Amur, Wenjun Bao, Catalin C Barbacioru, Vincent Bertholet, Xiaoxi Megan Cao, Tzu-Ming Chu, Patrick J Collins, Xiao-hui Fan, Felix W Frueh, James C Fuscoe, Xu Guo, Jing Han, Damir Herman, Huixiao Hong, Ernest S Kawasaki, Quan-Zhen Li, Yuling Luo, Yunqing Ma, Nan Mei, Ron L Peterson, Raj K Puri, Richard Shippy, Zhenqiang Su, Yongming Andrew Sun, Hongmei Sun, Brett Thorn, Yaron Turpaz, Charles Wang, Sue Jane Wang, Janet A Warrington, James C Willey, Jie Wu, Qian Xie, Liang Zhang, Lu Zhang, Sheng Zhong, Russell D Wolfinger, Weida Tong

**Affiliations:** 1 National Center for Toxicological Research, US Food and Drug Administration, 3900 NCTR Road, Jefferson, AR 72079, USA; 2 Expression Analysis Inc., 2605 Meridian Parkway, Durham, NC 27713, USA; 3 University of Massachusetts Boston, Department of Physics, 100 Morrissey Boulevard, Boston, MA 02125, USA; 4 Z-Tech Corporation, an ICF International Company at NCTR/FDA, 3900 NCTR Road, Jefferson, AR 72079, USA; 5 Center for Drug Evaluation and Research, US Food and Drug Administration, 10903 New Hampshire Avenue, Silver Spring, MD 20993, USA; 6 Biogen Idec Inc., 5200 Research Place, San Diego, CA 92122, USA; 7 ViaLogy Inc., 2400 Lincoln Avenue, Altadena, CA 91001, USA; 8 SAS Institute Inc., SAS Campus Drive, Cary, NC 27513, USA; 9 Applied Biosystems, 850 Lincoln Centre Drive, Foster City, CA 94404, USA; 10 Eppendorf Array Technologies, rue du Séminaire 20a, 5000 Namur, Belgium; 11 Agilent Technologies Inc., 5301 Stevens Creek Boulevard, Santa Clara, CA 95051, USA; 12 Pharmaceutical Informatics Institute, Zhejiang University, Hangzhou 310027, China; 13 Affymetrix Inc., 3420 Central Expressway, Santa Clara, CA 95051, USA; 14 Center for Biologics Evaluation and Research, US Food and Drug Administration, 8800 Rockville Pike, Bethesda, MD 20892, USA; 15 National Center for Biotechnology Information, National Library of Medicine, National Institutes of Health, 8600 Rockville Pike, Bethesda, MD 20894, USA; 16 National Cancer Institute Advanced Technology Center, 8717 Grovemont Circle, Gaithersburg, MD 20877, USA; 17 University of Texas Southwestern Medical Center, 6000 Harry Hines Boulevard, Dallas, TX 75390, USA; 18 Panomics Inc., 6519 Dumbarton Circle, Fremont, CA 94555, USA; 19 Novartis Institutes for Biomedical Research, 250 Massachusetts Avenue, Cambridge, MA 02139, USA; 20 GE Healthcare, 7700 S River Parkway, Tempe, AZ 85284, USA; 21 UCLA David Geffen School of Medicine, Transcriptional Genomics Core, Cedars-Sinai Medical Center, 8700 Beverly Boulevard, Los Angeles, CA 90048, USA; 22 Ohio Medical University, 3000 Arlington Avenue, Toledo, OH 43614, USA; 23 CapitalBio Corporation, 18 Life Science Parkway, Changping District, Beijing 102206, China; 24 Solexa Inc., 25861 Industrial Boulevard, Hayward, CA 94545, USA; 25 University of Illinois at Urbana-Champaign, Department of Bioengineering, 1304 W. Springfield Avenue, Urbana, IL 61801, USA

## Abstract

**Background:**

Reproducibility is a fundamental requirement in scientific experiments. Some recent publications have claimed that microarrays are unreliable because lists of differentially expressed genes (DEGs) are not reproducible in similar experiments. Meanwhile, new statistical methods for identifying DEGs continue to appear in the scientific literature. The resultant variety of existing and emerging methods exacerbates confusion and continuing debate in the microarray community on the appropriate choice of methods for identifying reliable DEG lists.

**Results:**

Using the data sets generated by the MicroArray Quality Control (MAQC) project, we investigated the impact on the reproducibility of DEG lists of a few widely used gene selection procedures. We present comprehensive results from inter-site comparisons using the same microarray platform, cross-platform comparisons using multiple microarray platforms, and comparisons between microarray results and those from TaqMan – the widely regarded "standard" gene expression platform. Our results demonstrate that (1) previously reported discordance between DEG lists could simply result from ranking and selecting DEGs solely by statistical significance (*P*) derived from widely used simple *t*-tests; (2) when fold change (FC) is used as the ranking criterion with a non-stringent *P*-value cutoff filtering, the DEG lists become much more reproducible, especially when fewer genes are selected as differentially expressed, as is the case in most microarray studies; and (3) the instability of short DEG lists solely based on *P*-value ranking is an expected mathematical consequence of the high variability of the *t*-values; the more stringent the *P*-value threshold, the less reproducible the DEG list is. These observations are also consistent with results from extensive simulation calculations.

**Conclusion:**

We recommend the use of FC-ranking plus a non-stringent *P *cutoff as a straightforward and baseline practice in order to generate more reproducible DEG lists. Specifically, the *P*-value cutoff should not be stringent (too small) and FC should be as large as possible. Our results provide practical guidance to choose the appropriate FC and *P*-value cutoffs when selecting a given number of DEGs. The FC criterion enhances reproducibility, whereas the *P *criterion balances sensitivity and specificity.

## Background

A fundamental step in most microarray experiments is determining one or more short lists of differentially expressed genes (DEGs) that distinguish biological conditions, such as disease from health. Challenges regarding the reliability of microarray results have largely been founded on the inability of researchers to replicate DEG lists across highly similar experiments. For example, Tan *et al*. [1] found only four common DEGs using an identical set of RNA samples across three popular commercial platforms. Independent studies by the groups of Ramalho-Santos [2] and Ivanova [3] of stem cell-specific genes using the same Affymetrix platform and similar study design found a disappointing six common DEGs among about 200 identified in each study [4]. A comparative neurotoxicological study by Miller *et al*. [5] using the same set of RNA samples found only 11 common DEGs among 138 and 425, respectively, from Affymetrix and CodeLink platforms. All these studies ranked genes by *P*-value from simple *t*-tests, used a *P *threshold to identify DEG lists, and applied the concept of the Percentage of Overlapping Genes (POG), or the Venn diagram, between DEG lists as the measure of reproducibility.

Criticism of and concerns about microarrays continue to appear in some of the most prestigious scientific journals [6-10], leading to a growing negative perception regarding microarray reproducibility, and hence reliability. However, in reanalyzing the data set of Tan *et al*. [1], Shi *et al*. [11] found that cross-platform concordance was markedly improved when either simple fold change (FC) or Significance Analysis of Microarrays (SAM) [12] methods were used to rank order genes before determining DEG lists. The awareness that microarray reproducibility is sensitive to how DEGs are identified was, in fact, a major motivator for the MicroArray Quality Control (MAQC) project [11,13,14].

Several plausible explanations and solutions have been proposed to interpret and address the apparent lack of reproducibility and stability of DEG lists from microarray studies. Larger sample sizes [15]; novel, microarray-specific statistical methods [16]; more accurate array annotation information by mapping probe sequences across platforms [1,17]; eliminating absent call genes from data analysis [11,18,19]; improving probe design to minimize cross-hybridization [17]; standardizing manufacturing processes [1]; and improving data quality by fully standardizing sample preparation and hybridization procedures are among the suggestions for improvement [20].

The MAQC study [13] was specifically designed to address these previously identified sources of variability in DEG lists. Two distinct RNA samples, Stratagene Universal Human Reference RNA (*i.e*., MAQC sample A) and Ambion Human Brain Reference RNA (*i.e*., MAQC sample B), with thousands of differentially expressed genes, were prepared in sufficient quantities and distributed to three different laboratories for each of the five different commercial whole genome microarray platforms participating in the study. For each platform, each sample was analyzed using five technical replicates with standardized procedures for sample processing, hybridization, scanning, data acquisition, data preprocessing, and data normalization at each site. The probe sequence information was used to generate a stringent mapping of genes across the different platforms and 906 genes were further analyzed with TaqMan^® ^assays using the same RNA samples.

In addition to assessing the technical performance of different microarray platforms, the MAQC study also discussed the idea of using fold-change ranking along with a non-stringent *P*-value cutoff for selecting DEGs [13,21]. However, a lot of detailed results have not been formally published to support the idea [22]. The MAQC project, while positively received by the community [23-27], also stimulated criticism from the statistical community about appropriate ways of identifying DEGs [22,23,27-33].

To help the microarray community better understand the issue at debate and move forward, in this study, we conducted a careful analysis of these MAQC data sets, along with numerical simulations and mathematical arguments. We demonstrate that the reported lack of reproducibility of DEG lists can be attributed in large part to identifying DEGs from simple *t*-tests without consideration of FC. The finding holds for intra-laboratory, inter-laboratory, and cross-platform comparisons independent of sample pairs and normalization methods, and is increasingly apparent with decreasing number of genes selected.

As a basic procedure for improving reproducibility while balancing specificity and sensitivity, choosing genes using a combination of FC-ranking and *P *threshold was investigated. This joint criterion results in DEG lists with much higher POG, commensurate with better reproducibility, than lists generated by *t*-test *P *alone, even when selecting a relatively small numbers of genes. An FC criterion explicitly incorporates the measured quantity to enhance reproducibility, whereas a *P *criterion incorporates control of sensitivity and specificity. The results increase our confidence in the reproducibility of microarray studies while supporting a need for caution in the use of inferential statistics when selecting DEGs. While numerous more advanced statistical modeling techniques have been proposed and compared for selecting DEGs [16,34,35], the primary objectives here are to explain that the primary reason for microarray reproducibility concerns is failure to include an FC criterion during gene selection, and to recommend a simple and straightforward approach concurrently satisfying statistical and reproducibility requirements. It should be stressed that robust methods are needed to meet stringent clinical requirements for reproducibility, sensitivity and specificity of microarray applications in, for example, clinical diagnostics and prognostics.

## Results

The POG for a number of gene selection scenarios employing *P *and/or FC are compared and a numerical example (see side box) is provided that shows how the simple *t*-test, when sample size is small, results in selection of different genes purely by chance. While the data generate from the MAQC samples A and B lack biological variability, the results are supported by the toxicogenomic data of Guo *et al*. [21] While *P *could be computed from many different statistical methods, for simplicity and consistency, throughout this article *P *is calculated with the two-tailed *t*-test that is widely employed in microarray data analysis.

### Inter-site concordance for the same platform

**Figure 1:**
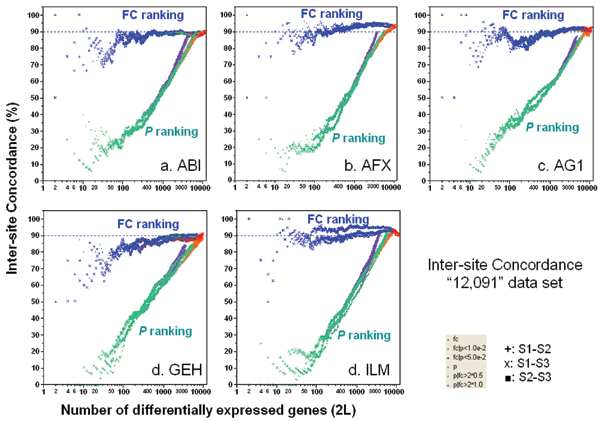
Concordance for inter-site comparisons. Each panel represents the POG results for a commercial platform of inter-site consistency in terms of DEGs between samples B and A. For each of the six gene selection methods, there are three possible inter-site comparisons: S1–S2, S1–S3, and S2–S3 (S = Site). Therefore, each panel consists of 18 POG lines that are colored based on gene ranking/selection method. Results shown here are based on the entire set of "12,091" genes commonly mapped across the microarray platforms without noise (absent call) filtering. POG results are improved when the analyses are performed using the subset of genes that are commonly detectable by the two test sites, as shown in Figure 2. The x-axis represents the number of selected DEGs, and the y-axis is the percentage (%) of genes common to the two gene lists derived from two test sites at a given number of DEGs.

Figure 1 gives plots of inter-site POG versus the number of genes selected as differentially expressed. Since there are three possible inter-site comparisons (S1–S2, S1–S3, and S2–S3, where S = Site) and six gene selection methods (see Methods), there are 18 POG lines for each platform. Figure 1 shows that inter-site reproducibility in terms of POG heavily depends on the number of chosen differential genes and the gene ranking criterion: Gene selection using FC-ranking gives consistently higher POG than *P*-ranking. The POG from FC-ranking is near 90% for as few as 20 genes for most platforms, and remains at this high inter-site concordance level as the number of selected genes increases. In contrast, the POG from *P*-ranking is in the range of 20–40% for as many as 100 genes, and then asymptotically approaches 90% only after several thousand genes are selected.

**Figure 2:**
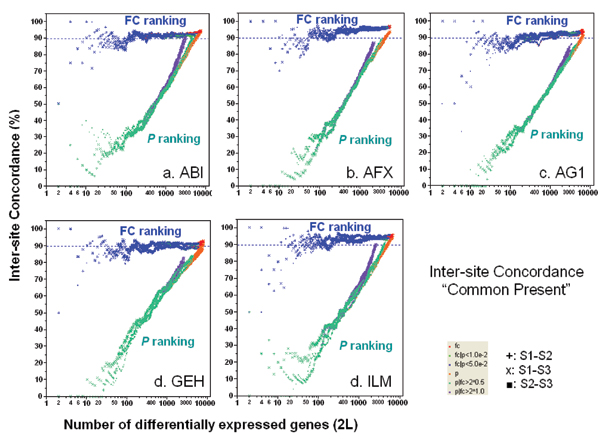
Concordance for inter-site comparisons based on genes commonly detectable by the two test sites compared. Each panel represents the POG results for a commercial platform of inter-site consistency in terms of DEGs between samples B and A. For each of the six gene selection methods, there are three possible inter-site comparisons: S1–S2, S1–S3, and S2–S3. Therefore, each panel consists of 18 POG lines that are colored based on gene ranking/selection method. The x-axis represents the number of selected DEGs, and the y-axis is the percentage (%) of genes common to the two gene lists derived from two test sites at a given number of DEGs.

The POG is higher when the analyses are limited to the genes commonly detected ("Present" in the majority of replicates for each sample) by both test sites under comparison (Figure 2). In addition to a slight increase (2–3%), the inter-site POG lines after noise filtering are more stable than those before noise filtering, particularly for ABI, AG1, and GEH. Furthermore, differences between the three ILM test sites are further decreased after noise filtering, as seen from the convergence of the POG for S1–S2, S1–S3, and S2–S3 comparisons. Importantly, noise filtering does not change either the trend or magnitude of the higher POG graphs for FC-ranking compared with *P*-ranking.

**Figure 3:**
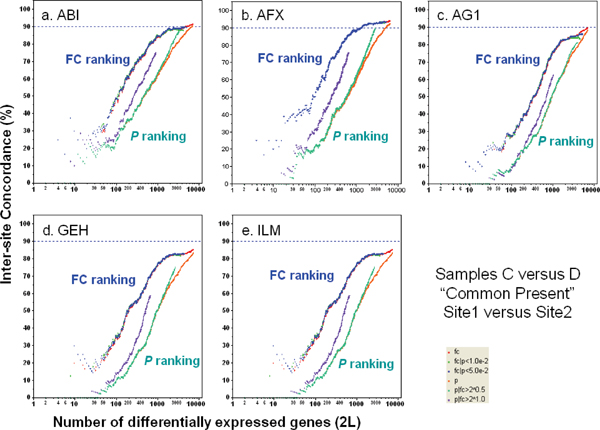
Concordance for inter-site comparison with samples C and D. The largest fold change between samples C and D is small (three-fold). For each platform, DEG lists from sites 1 and 2 are compared. Analyses are performed using the subset of genes that are commonly detectable by the two test sites.

Inter-site concordance for different FC- and *P*-ranking criteria were also calculated for other MAQC sample pairs having much smaller differences than for sample A versus sample B, and correspondingly lower FC. In general, POG is much lower for other sample pairs regardless of ranking method and ranking order varies more greatly, though FC-ranking methods still consistently give a higher POG than *P*-ranking methods. Figure 3 gives the plots of POG for Sample C versus Sample D, a 3:1 and 1:3 (A:B) mixture, respectively[13,36], for all inter-site comparisons.

The substantial difference in inter-site POG shown in Figures 1 and 2 is a direct result of applying different gene selection methods to the same data sets, and clearly depicts how perceptions of inter-site reproducibility can be affected for any microarray platform. While the emphasis here is on reproducibility in terms of POG, in practice, this criterion must be balanced against other desirable characteristics of gene lists, such as specificity and sensitivity (when the truth is binary) or mean squared error (when the truth is continuous), considerations that that are discussed further in later sections.

### Cross-platform concordance

**Figure 4:**
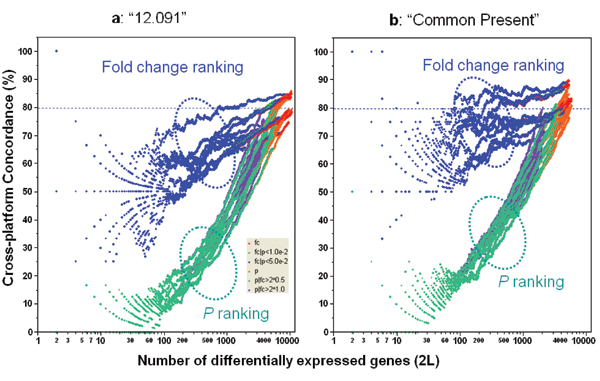
Concordance for cross-platform comparisons. Panel a: Based on the data set of "12,091" genes (without noise filtering); Panel b: Based on subsets of genes commonly detected ("Present") by two platforms. For each platform, the data from test site1 are used for cross-platform comparison. Each POG line corresponds to comparison of the DEGs from two microarray platforms using one of the six gene selection methods. There are ten platform-platform comparison pairs, resulting in 60 POG lines for each panel. The x-axis represents the number of selected DEGs, and the y-axis is the percentage (%) of genes common to the two gene lists derived from two platforms at a given number of DEGs. POG lines circled by the blue oval are from FC based gene selection methods with or without a *P *cutoff, whereas POG lines circled by the teal oval are from *P *based gene selection methods with or without an FC cutoff. Shown here are results for comparing sample B and sample A.

Figure 4 shows the substantial effect that FC- and *P*-ranking based gene selection methods have on cross-platform POG. Similar to inter-site comparisons, *P*-ranking results in much lower cross-platform POG than FC-ranking. When FC is used to rank DEGs from each platform, the cross-platform POG is around 70–85%, depending on the platform pair. The platforms themselves contribute about 15% differences in the cross-platform POG, as seen from the spread of the blue POG lines. Noise filtering improves FC-ranking based cross-platform POG by about 5–10% and results in more stable POG when a smaller number of genes are selected (Figure 4b). Importantly, the relative differences between FC- and *P*-ranking methods remain the same after filtering.

### Concordance between microarray and TaqMan^® ^assays

**Figure 5:**
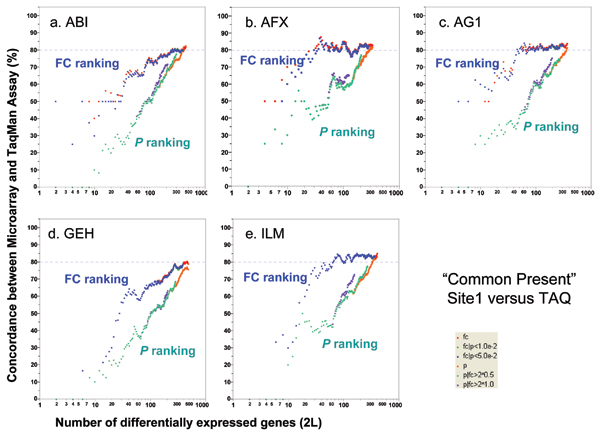
Concordance between microarray and TaqMan^® ^assays. Each panel represents the comparison of one microarray platform to TaqMan^® ^assays. For each microarray platform, the data from test site 1 are used for comparison to TaqMan^® ^assays. Each POG line corresponds to comparison of the DEGs from one microarray platform and those from the TaqMan^® ^assays using one of the six gene selection methods. The x-axis represents the number of selected DEGs, and the Y-axis is the percentage (%) of genes common to DEGs derived from a microarray platform and those from TaqMan^® ^assays. Shown here are results for comparing sample B and sample A using a subset of genes that are detectable by both the microarray platform and TaqMan^® ^assays. Results based on the entire set of 906 genes are provided in Figure 6.

**Figure 6:**
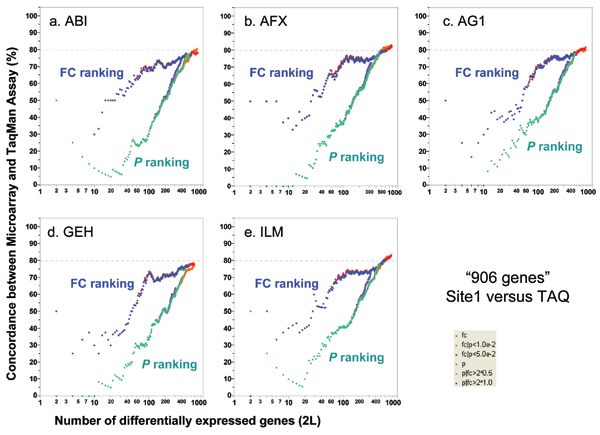
Concordance between microarray and TaqMan^® ^assays without noise-filtering. Each panel represents the comparison of one microarray platform to TaqMan^® ^assays. The x-axis represents the number of selected DEGs, and the y-axis is the percentage (%) of genes common to DEGs derived from a microarray platform and those from TaqMan^® ^assays. Shown here are results for comparing sample B and sample A using the entire set of 906 genes for which TaqMan^® ^assay data are available.

TaqMan^® ^real-time PCR assays are widely used to validate microarray results [37,38]. In the MAQC project, the expression levels of 997 genes randomly selected from available TaqMan^® ^assays have been quantified in the four MAQC samples [13,39]. Nine hundred and six (906) of the 997 genes are among the "12,091" set of genes found on all of the six genome-wide microarray platforms [13]. There are four technical replicates for each sample and the DEGs for TaqMan^® ^assays were identified using the same six gene selection procedures as those used for microarray data. The DEGs calculated from the microarray data are compared with DEGs calculated from TaqMan^® ^assay data. With noise filtering (*i.e*., focusing on the genes detected by both the microarray platform and TaqMan^® ^assays), 80–85% concordance was observed (Figure 5). Consistent with inter-site and cross-platform comparisons, POGs comparing microarray with TaqMan^® ^assays also show that ranking genes by FC results in markedly higher POG than ranking by *P *alone, especially for short gene lists. POG results without noise filtering (Figure 6) are some 5% lower but the notable differences in POG between the FC- and *P*-ranking are unchanged.

### Reproducibility of FC and t-statistic: different metrics for identifying differentially expressed genes (DEGs)

**Figure 7:**
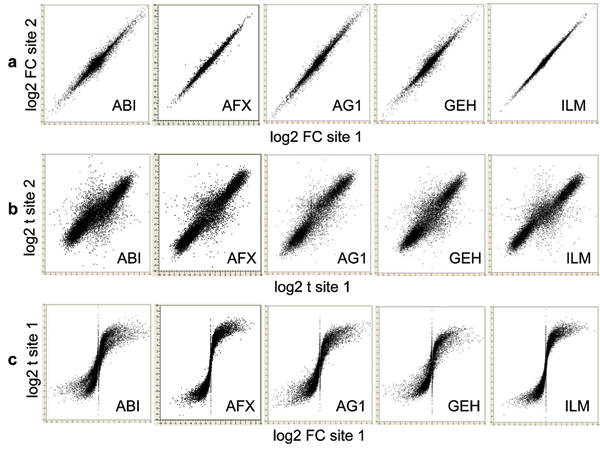
Inter-site reproducibility of log2 FC and log2 t-statistic. a: log2 FC of site 1 versus log2 FC of site 2; b: log2 t-statistic of test site 1 versus log2 t-statistic of test site 2; and c: log2 FC of test site 1 versus log2 t-statistic of test site 1. Shown here are results for comparing sample B and sample A for all "12,091" genes commonly probed by the five microarray platforms. The inter-site reproducibility of log2 FC (a) is much higher than that of log2 t-statistic (b). The relationship between log2 FC and log2 t-statistic from the same test site is non-linear and the correlation appears to be low (c).

**Figure 8:**
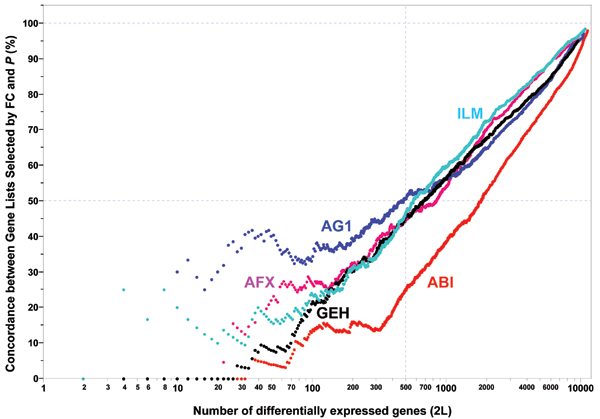
Concordance between FC and *P *based gene ranking methods ("12,091 genes"; test site 1). Each POG line represents a platform using data from its first test site. The x-axis represents the number of selected DEGs, and the y-axis is the percentage (%) of genes common in the DEGs derived from FC- and *P*-ranking. Shown here are results for comparing sample B and sample A for all "12,091" genes commonly probed. When a smaller number of genes (up to a few hundreds or thousands) are selected, POG for cross selection method comparison (FC vs. *P*) is low. For example, there are only about 50% genes in common for the top 500 genes selected by FC and *P *separately, indicating that FC and *P *rank order DEGs dramatically differently. When the number of selected DEGs increases, the overlap between the two methods increases, and eventually approach to 100% in common, as expected. The low concordance between FC- and *P*-based gene ranking methods is not unexpected considering their different definitions and low correlation (Figure 7c).

Figure 7 shows that the inter-site reproducibility of log2 FC (panel a) is much higher than that of log2 t-statistic (panel b). In addition, the relationship between log2 FC and log2 t-statistic from the same test site is non-linear and the correlation appears to be low (panel c). We see similar results when data from different microarray platforms are compared to each other or when microarray data are compared against TaqMan^® ^assay data (results not shown). The differences between the reproducibility of FC and *t*-statistic observed here are consistent with the differences between POG results in inter-site (Figures 1, 2, 3), cross-platform (Figure 4), and microarray versus TaqMan^® ^assay (Figures 5 and 6) comparisons. The nonlinear relationship between log2 FC and log2 t-statistic (Figure 7c) leads to low concordance between the list of DEGs derived from FC-ranking and the list derived from t-statistic (*P*) ranking (Figure 8); an expected outcome due to the different emphases of FC and *P*.

### Joint FC and *P *rule illustrated with a volcano plot: ranking by FC, not by *P*

**Figure 9:**
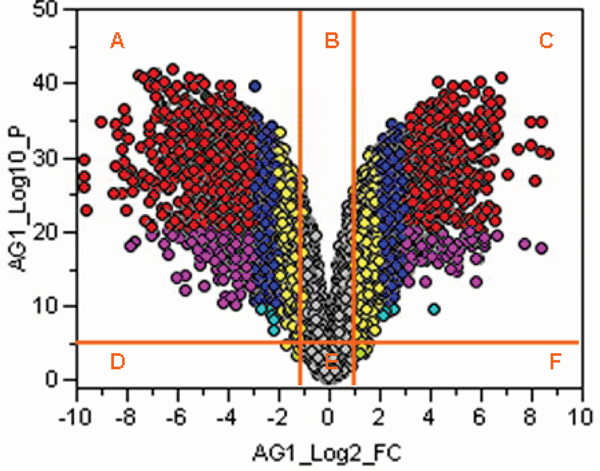
Volcano plot illustration of joint FC and *P *gene selection rule. Genes in sectors A and C are selected as differentially expressed. The colors correspond to the negative log_10 _*P *and log_2 _fold change values: Red: 20 < -log_10 _*P *< 50 and 3 < log_2 _fold < 9 or -9 < log_2 _fold < -3. Blue: 10 < -log_10 _*P *< 50 and 2 < log_2 _fold < 3 or -3 < log_2 _fold < -2. Yellow: 4 < -log_10 _*P *< 50 and 1 < log_2 _fold < 2 or -2 < log_2 _fold < -1. Pink : 10 < -log_10 _*P *< 20 and 3 < log_2 _fold or log_2 _fold < -3. Light blue: 4 < -log_10 _*P *< 10 and 2 < log_2 _fold or log_2 _fold < -2. Light green: 2 < -log_10 _*P *< 4 and 1 < log_2 _fold or log_2 _fold < -1. Gray)

Figure 9 is a volcano plot depicting how a joint FC and *P *rule works in gene selection. It uses the MAQC Agilent data, and plots negative log10 *P *on the y-axis versus log2 FC on the x-axis. A joint rule chooses genes that fall in the upper left and upper right sections of the plot (sections A and C of Figure 9). Other possible cutoff rules for combining FC and *P *are apparent, but are precluded from inclusion due to space. An important conclusion from this study is that genes should be ranked and selected by FC (x-axis) with a non-stringent *P *threshold in order to generate reproducible lists of DEGs. The more stringent the *P*-value threshold, the less reproducible the DEG list is. Our results provide practical guidance to choose the appropriate FC and *P*-value cutoffs in using the "volcano plots" to select DEGs.

### Concordance using other statistical tests

**Figure 10:**
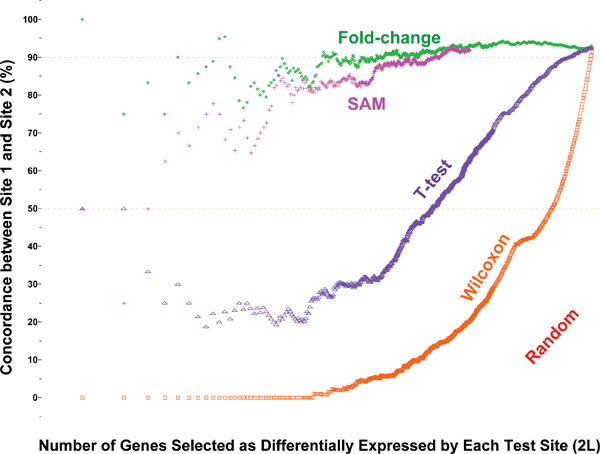
Inter-site concordance based on FC, t-test, Wilcoxon rank-sum test, and SAM. Affymetrix data on samples A and B from site 1 and site 2 for the "12,091" commonly mapped genes were used[13]. No flagged ("Absent") genes were excluded in the analysis. For the Wilcoxon rank-sum tests, there were many ties, *i.e*., many genes exhibited the same level of statistical significance because of the small sample sizes (five replicates for each group). The tied genes from each test site were broken (ranked) by random ordering. Concordance between genes selected completely by random choice is shown in red and reaches 50% when all candidate genes are declared as differentially expressed; the other 50% genes are in opposite regulation directions. SAM improves inter-site reproducibility compared to t-test, and approaches, but does not exceed that of fold-change.

Numerous different statistical tests including rank tests (*e.g*., Wilcoxon rank-sum test) and shrunken t-tests (*e.g*., SAM) have been used for the identification of DEGs. Although this work is not intended to serve as a comprehensive performance survey of different statistical procedures, we set out to briefly examine a few examples due to their popularity. Figure 10 shows the POG results of several commonly used approaches including FC-ranking, t-test statistic, Wilcoxon rank-sum test, and SAM using AFX site-site comparison as an example[13]. The POG by SAM (pink line), although greatly improved over that of simple t-test statistic (purple line), approached, but did not exceed, the level of POG based on FC-ranking (green line). In addition, the small numbers of replicates in this study rendered many ties in the Wilcoxon rank statistic, resulting in poor inter-site concordance in terms of rank-order of the DEGs between the two AFX test sites. Similar findings (data not shown) were observed using the toxicogenomics data set of Guo *et al*. [21].

### Gene selection in simulated datasets

The MAQC data, like data from actual experiments, allows evaluation of DEG list reproducibility, but not of truth. Statistics are used to estimate truth, often in terms of sensitivity and specificity, but the estimates are based on assumptions about data variance and error structure that are also unknown. Simulations where truth can be specified *a priori *are useful to conduct parametric evaluations of gene selection methods, and true false positives and negatives are then known. However, results are sensitive to assumptions regarding data structure and error that for microarrays remains poorly characterized.

**Figure 11:**
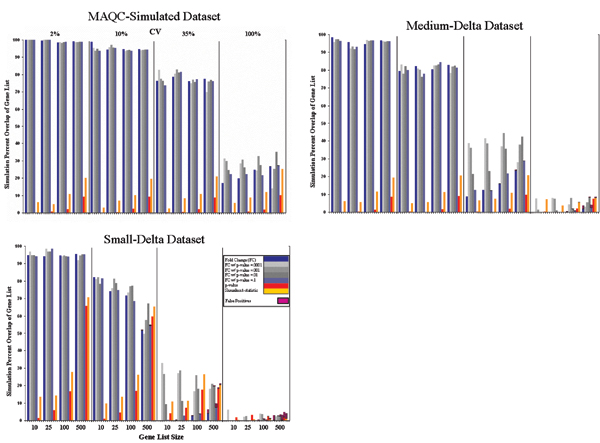
Gene selection and percentage of agreement in gene lists in simulated data sets. Illustrations of the effect of biological context, replicate CV distribution, gene list size, and gene selection rules/methods on the reproducibility of gene lists using simulated microarray data. In some sense, these three graphs represent some extremes as well as typical scenarios in differential expression assays. However, FC sorting with low *P *thresholds (0.001 or 0.0001; light and medium gray boxes) consistently performed better overall than the other rules, even when FC-ranking or *P*-ranking by itself did not perform as well.

Figure 11 gives POG versus the number of genes for three simulated data sets (MAQC-simulated set, Small-Delta simulated set, and Medium-Delta simulated set, see Methods) that were prepared in order to compare the same gene selection methods as the MAQC data. The MAQC-simulated set was created to emulate the magnitude and structure of differential expression observed between the actual MAQC samples A and B (*i.e*., thousands of genes with FC > 2). By comparison, the Small-Delta simulated data set had only 50 significant genes with FC > 2 and most genes had FC < 1.3. The Medium-Delta data set had FC profiles in between.

For the MAQC-simulated data, either FC-ranking or FC-ranking combined with any of the *P *threshold resulted in markedly higher POG than any *P*-ranking method, regardless of gene list length and coefficient of variation (CV) of replicates. The POG is ~100%, ~95%, and ~75%, for replicate CV values of 2%, 10%, and 35% CV, respectively, decreasing to about 20–30% with an exceedingly high (100%) CV. In contrast, POG from *P*-ranking alone varies from a few percent to only ~10% when 500 genes are selected.

For the Medium- and Small-Delta simulated data sets, differences start to emerge between using FC alone and FC with *P *cutoff. From Figure 11, when variances in replicates become larger (CV > 10%), the reproducibility is greatly enhanced using FC-ranking with a suitable *P *cutoff versus FC or *P *by themselves. In addition, when variances are small (CV ≤ 10%), the reproducibility is essentially the same for FC with *P *or without. What is clear is that *P *by itself did not produce the most reproducible DEG list under any simulated condition.

Although *P*-ranking generally resulted in very low POG, a false positive was rarely produced, even for a list size of 500 (data not shown). Thus, the *P *criterion performed as expected, and identified mostly true positives. Unfortunately, the probability of selection of the same true positives with a fixed *P *cutoff in a replicated experiment appears small due to variation in the *P *statistic itself (see inset). FC-ranking by itself resulted in a large number of false positives with a large number of genes for the Medium and Small-Delta sets when genes with small FCs are selected as differentially expressed. These false positives were greatly reduced to the same level as for the *P*-ranking alone when FC-ranking was combined with a *P*-cutoff.

### Variability of the two-sample t-statistic

In a two-sample t-test comparing the mean of sample A to the mean of sample B, the t-statistic is given as follows:

t=X¯B−X¯ASp2nB+Sp2nA

where ${\bar{X}}_{A}$ is the average of the log2 expression levels of sample A with *n*_*A *_replicates, and ${\bar{X}}_{B}$ is the average of the log2 expression levels of sample B with *n*_*B *_replicates, and *S*_*p*_^2 ^= (*SS*_*A*_+*SS*_*B*_)/(*n*_*A*_+*n*_*B*_-*2*) is the pooled variance of samples A and B, and *SS *denotes the sum of squared errors. The numerator of the t-statistic is the fold-change (FC) in log2 scale and represents the signal level of the measurements (*i.e*., the magnitude of the difference between the expression levels of sample A and sample B). The denominator represents the noise components from the measurements of samples A and B. Thus, the t-statistic represents a measure of the signal-to-noise ratio. Therefore, the FC and the t-statistic (*P*) are two measures for the differences between the means of sample A and sample B. The t-statistic is intrinsically less reproducible than FC when the variance is small.

Assume that the data are normally distributed, the variances of samples A and B are equal (*σ*^2^), the numbers of replicates in samples A and B are equal (*n = n*_*A *_= *n*_*B*_), and that there is a real difference in the mean values between samples A and B, *d *(the true FC in log2 scale). Then the t-statistic has a non-central t-distribution with non-central parameter

$$\delta =\left(d/\sigma \right)\left(\sqrt{n/2}\right),$$

and the mean and variance of the *t*-statistic (Johnson and Kotz, 1970) are

$$\begin{array}{cc} E\left(t\right)={\left(\frac{1}{2}\nu \right)}^{\frac{1}{2}}\frac{\Gamma \left(\frac{1}{2}\left(\nu -1\right)\right)}{\Gamma \left(\frac{1}{2}\nu \right)}\delta , & Var\left(t\right)=\frac{\nu }{\nu -2}+\left(\frac{\nu }{\nu -2}-{\left[{\left(\frac{1}{2}\nu \right)}^{\frac{1}{2}}\frac{\Gamma \left(\frac{1}{2}\left(\nu -1\right)\right)}{\Gamma \left(\frac{1}{2}\nu \right)}\right]}^{2}\right)\delta^{2} \end{array}$$

where *v *= (*2n-2*) and is the degrees of freedom of the non-central t-distribution. When *d *= 0 (the two means are equal), then the t-statistic has a t-distribution with mean *E*(*t*) = 0 and *Var*(*t*) = *v*/(*v*-2). The variance of the t-statistic depends on the sample size *n*, the magnitude of the difference between the two means *d*, and the variance *σ*^2^. On the other hand, the variance of the mean difference for the FC is (2/*n*)*σ*^2^. That is, the variance of the FC depends only on the sample size *n *and the variance *σ*^2^, regardless of the magnitude of the difference *d *between the two sample means.

In an MAQC data set, a typical sample variance for the log2 expression levels is approximately *σ*^2 ^= 0.15^2^. With *n *= 5, the standard deviation of the FC (in log2 scale) is 0.09. For a differentially expressed gene with a 4-fold change between 5 replicates of sample A and 5 replicates of sample B, *d *= 2 and the t-values have a non-central t-distribution with (*ν *= *n*_A_+*n*_B_-2) = 8 degrees of freedom and *δ *= 21.08. From the equations above, the mean and the variance of the t-values are E(*t*) = 23.35 and Var(*t*) = 6.96^2^. Within two standard deviations the expected value of the t-value ranges from 9.43 (= 23.35-2 × 6.96) to 37.27 (= 23.35+2 × 6.96), corresponding to *P*s from 1 × 10^-5 ^to 3 × 10^-10^, based on the Student's two-sided t-test with 8 degrees of freedom. In contrast, when *n *= 5 the standard deviation of the FC (in log2 scale) is 0.09. The expected value of the FC ranges only from 3.53 (= 2^1.82^) to 4.53 (= 2^2.18^) within two standard deviations. In this case, this gene would be selected as differentially expressed using either a FC cutoff of 3.5 or a *P *cutoff of 1 × 10^-5^. On the other hand, for a gene with a 2-fold change (*d *= 1), the t-statistic has a non-central t-distribution with *δ *= 10.54. The mean and the variance of the t-statistic are E(*t*) = 11.68 and Var(*t*) = 3.62^2 ^with a corresponding *P *of 3 × 10^-6 ^at t = 11.68. Using the same *P *cutoff, 1 × 10^-5^, this gene is likely to be selected with the probability greater than 0.5. For the FC criterion, the expected value of the FC ranges from 1.76 (= 2^0.82^) to 2.26 (= 2^1.18^). Using the same FC cutoff of 3.5, this gene is very unlikely to be selected. Thus, the top ranked gene list based on the FC is more reproducible than the top ranked gene list based on the *P*. The top ranked genes selected by a *P *cutoff may not be reproducible between experiments although both lists may contain mostly differentially expressed genes.

Reference: N. Johnson and S. Kotz (1970). Continuous Univariate Distributions – 2. Houghton Mifflin, Boston.

## Discussion

A fundamental requirement in microarray experiments is that the identification of DEG lists must be reproducible if the data and scientific conclusion from them are to be credible. DEG lists are normally developed by rank-ordering genes in accordance with a suitable surrogate value to represent biological relevance, such as the magnitude of the differential expression (*i.e*., FC) or the measure of statistical significance (*P*) of the expression change, or both. The results show that concurrent use of both FC-ranking and *P*-cutoff as criteria to identify biological relevant genes can be essential to attain reproducible DEG lists across laboratories and platforms.

A decade since the microarray-generated differential gene expression results of Schena *et al*[40] and Lockhart *et al*[41] were published, microarray usage has become ubiquitous. Over this time, many analytical techniques for identifying DEGs have been introduced and used. Early studies predominantly relied on the magnitude of differential expression change in experiments done with few if any replicates, with an FC cutoff typically of two used to reduce false positives. Mutch *et al*[42] recommended using intensity-dependent FC cutoffs to reduce biased selection of genes with low expression.

Gene selection using statistical significance estimates became more prevalent during the last few years as studies with replicates became possible. Incorporation of a t-statistic in gene selection was intended to compensate for the heterogeneity of variances of genes [43]. Haslett *et al*. [44] employed stringent values of both FC and *P *to determine DEGs. In recent years, there has been an increasing tendency to use *P*-ranking for gene selection. Kittleson *et al*. [45] selected genes with a FC cutoff of two and a very restrictive Bonferroni corrected *P *of 0.05 in a quest for a short list of true positive genes. Tan *et al*. [46] used *P *to rank genes. Correlation coefficient, which behaves similarly to the t-statistic, has also been widely used as a gene selection method in the identification of signature genes for classification purposes [15,47,48].

New and widely employed methods have appeared in recent years and implicitly correct for the large variance in the t-statistic that results when gene variance is estimated with a small number of samples. Allison *et al*. [16] collectively described these methods as "variance shrinkage" approaches. They include the popular permutation-based "SAM" procedure [5,12,49,50], Bayesian-based approaches [43,51] and others [52]. Qin *et al*. [34] compared several variance shrinkage methods with a simple t-statistic and FC for spike-in gene identification on a two-color platform, concluding that all methods except *P *performed well. All these methods have the effect of reducing a gene's variance to be between the average for the samples, and the average over the arrays.

In some cases, however, the use of FC for gene selection was criticized and entirely abandoned. For example, Callow *et al*. [53], using *P *alone for identifying DEGs, concluded that *P *alone eliminated the need for filtering low intensity spots because the t-statistic is uniformly distributed across the entire intensity range. Reliance on *P *alone to represent a gene's FC and variability in gene selection has become commonplace. Norris and Kahn [54] describe how false discovery rate (FDR) has become so widely used as to constitute a standard to which microarray analyses are held. However, FDR usually employs a shrunken t-statistic and genes are ranked and selected similar to *P *(see Figure 11).

Prior to MAQC, Irizarry *et al*. [55] compared data from five laboratories and three platforms using the CAT plots that are essentially the same as the POG graphs used in our study. Lists of less than 100 genes derived from FC-ranking showed 30 to 80% intra-site, inter-site, and inter-platform concordance. Interestingly, important disagreements were attributable to a small number of genes with large FC that they posit resulted from a laboratory effect due to inexperienced technicians and sequence-specific effects where some genes are not correctly measured.

Exactly how to best employ FC with *P *to identify genes is a function of both the nature of the data, and the inevitable tradeoff between sensitivity and specificity that is familiar across research, clinical screening and diagnostics, and even drug discovery. But how the tradeoff is made depends on the application. Fewer false negatives at the cost of more false positives may be desirable when the application is identifying a few hundred genes for further study, and FC-ranking with a non-stringent *P *value cutoff from a simple t-test could be used to eliminate some noise. The gene list can be further evaluated in terms of gene function and biological pathway data, as illustrated in Guo *et al*. [21] for toxicogenomic data. Even for relatively short gene lists, FC-ranking together with a non-stringent *P *cutoff should result in reproducible lists. In addition, DEG lists identified by the ranking of FC is much less susceptible to the impact of normalization methods. In fact, global scaling methods (*e.g*., median- or mean-scaling) do not change the relative ranking of genes based on FC; they do, however, impact gene ranking by *P*-value [21].

The tradeoffs between reproducibility, sensitivity, and specificity become pronounced when genes are selected by *P *alone without consideration of FC, especially when a stringent *P *cutoff is used to reduce false positives. When sample numbers are small, any gene's t-statistic can change considerably in repeated studies within or across laboratories or across platforms. Each study can select different significant genes, purely by chance. It is entirely possible that separately determined lists will have a small proportion of common genes even while each list comprises mostly true positives. This apparent lack of reproducibility of the gene lists is an expected outcome of statistical variation in the t-statistic for small numbers of sample replicates. In other words, each study fails to produce some, but not all, of the correct results. The side box provides a numerical example of how gene list discordance can result from variation in the t-statistic across studies. Decreasing the *P *cutoff will increase the proportion of true positives, but also diminish the number of selected genes, diminish genes common across lists, and increase false negatives. Importantly, selecting genes based on a small *P *cutoff derived from a simple t-test without consideration of FC renders the gene list non-reproducible.

Additional insight is gained by viewing gene selection from the perspective of the biologist ultimately responsible for interpreting microarray results. Statistically speaking, a microarray experiment tests 10,000 or more null hypotheses where essentially all genes have non-zero differential expression. Statistical tests attempt to account for an unknowable error structure, in order to eliminate the genes with low probability of biological relevance. To the biologist, however, the variance of a gene with a large FC in one microarray study may be irrelevant if a similar experiment again finds the gene to have a large FC; the second experiment would probably be considered a validating reproduction. This conclusion would be reasonable since the gene's *P *depends on a poor estimate of variance across few samples, whereas a repeated FC measurement is tangible reproducibility which tends to increase demonstrably with increasing FC. The biological interpreter can also consider knowledge of gene function and biological pathways before finally assigning biological relevance, and will be well aware that either *P *or FC is only another indicator regarding biological significance.

This study shows that genes with smaller expression fold changes generated from one platform or laboratory are, in general, less reproducible in another laboratory with the same or different platforms. However, it should be noted that genes with small fold changes may be biologically important [56]. When a fixed FC cutoff is chosen, sensitivity could be sacrificed for reproducibility. Alternatively, when a high *P *cutoff (or no *P *cutoff) is used, specificity could be sacrificed for reproducibility. Ultimately, the acceptable trade-off is based on the specific question being asked or the need being addressed. When searching for a few reliable biomarkers, high FC and low *P *cutoffs can be used to produce a highly specific and reproducible gene list. When identifying the components of genetic networks involved in biological processes, a lower FC and higher *P *cutoff can be used to identify larger, more sensitive but less specific, gene lists. In this case, additional biological information about putative gene functions can be incorporated to identify reliable gene lists that are specific to the biological process of interest.

Truly differentially expressed genes should be more likely identified as differentially expressed by different platforms and from different laboratories than those genes with no differential expression between sample groups. In the microarray field, we usually do not have the luxury of knowing the "truth" in a given study. Therefore, it is not surprising that most microarray studies and data analysis protocols have not been adequately evaluated against the "truth". A reasonable surrogate of such "truth" could be the consensus of results from different microarray platforms, from different laboratories using the same platform, or from independent methods such as TaqMan^® ^assays, as we have extensively explored in this study.

The fundamental scientific requirement of reproducibility is a critical dimension to consider along with balancing specificity and sensitivity when defining a gene list. Irreproducibility would render microarray technology generally, and any research result, specifically, vulnerable to criticism. New methods for the identification of DEGs continue to appear in the scientific literature. These methods are typically promoted in terms of improved sensitivity (power) while retaining nominal rates of specificity. However, reproducibility is seldom emphasized.

## Conclusion

The results show that selecting DEGs based solely on *P *from a simple t-test most often predestines a poor concordance in DEG lists, particularly for small numbers of genes. In contrast, using FC-ranking in conjunction with a non-stringent *P*-cutoff results in more concordant gene lists concomitant with needed reproducibility, even for fairly small numbers of genes. Moreover, enhanced reproducibility holds for inter-site, cross-platform, and between microarray and TaqMan^® ^assay comparisons, and is independent of platforms, sample pairs, and normalization methods. The results should increase confidence in the reproducibility of data produced by microarray technology and should also expand awareness that gene lists identified solely based on *P *will tend to be discordant. This work demonstrates the need for a shift from the common practice of selecting differentially expressed genes solely on the ranking of a statistical significance measure (*e.g*., t-statistic) to an approach that emphasizes fold-change, a quantity actually measured by microarray technology.

### Conclusions and recommendations

1. A fundamental step of microarray studies is the identification of a small subset of DEGs from among tens of thousands of genes probed on the microarray. DEG lists must be concordant to satisfy the scientific requirement of reproducibility, and must also be specific and sensitive for scientific relevance. A baseline practice is needed for properly assessing reproducibility/concordance alongside specificity and sensitivity.

2. Reports of DEG list instability in the literature are often a direct consequence of comparing DEG lists derived from a simple t-statistic when the sample size is small and variability in variance estimation is large. Therefore, the practice of using *P *alone for gene selection should be discouraged.

3. A DEG list should be chosen in a manner that concurrently satisfies scientific objectives in terms of inherent tradeoffs between reproducibility, specificity, and sensitivity.

4. Using FC and *P *together balances reproducibility, specificity, and sensitivity. Control of specificity and sensitivity can be accomplished with a *P *criterion, while reproducibility is enhanced with an FC criterion. Sensitivity can also be improved by better platforms with greater dynamic range and lower variability or by increased sample sizes.

5. FC-ranking should be used in combination with a non-stringent *P *threshold to select a DEG list that is reproducible, specific, and sensitive, and a joint rule is recommended as a baseline practice.

## Methods

### MAQC data sets

The MAQC data sets analyzed in this study are available from GEO under series accession number GSE5350. Analyses identified differentially expressed genes between the primary samples A (Stratagene Universal Human Reference RNA, Catalog #740000) and B (Ambion Human Brain Reference RNA, Catalog #6050) of the MAQC study. Analyses are additionally limited to data sets from the following five commercial genome-wide microarray platforms: ABI (Applied Biosystems), AFX (Affymetrix), AG1 (Agilent one-color), GEH (GE Healthcare), and ILM (Illumina), and to the subset of "12,091" genes commonly probed by them. TaqMan^® ^assay data for 906 genes are used to examine gene list comparability between microarrays and TaqMan^® ^assays. For more information about the MAQC project and the data sets, refer to Shi *et al *[13].

### Normalization methods

The following manufacturer's preferred normalization methods were used: quantile normalization for ABI and ILM, PLIER for AFX, and median-scaling for AG1 and GEH [13]. For quantile normalization (including PLIER), each test site is independently considered.

### Gene ranking (selection) rules

Six gene ranking (selection) methods were examined: (1) FC (fold change ranking); (2) FC_*P*0.05 (FC-ranking with *P *cutoff of 0.05); (3) FC_*P*0.01 (FC-ranking with *P *cutoff of 0.01); (4) *P (P*-ranking, simple t-test assuming equal variance); (5) *P*_FC2 (*P*-ranking with FC cutoff of 2); (6) *P*_FC1.4 (*P*-ranking with FC cutoff of 1.4). When a cutoff value (*e.g*., *P *< 0.05) is imposed for a ranking metric (*e.g*., FC), it is likely that the lists of candidate genes that meet the cutoff value may not be the same for the two test sites or two platforms as a result of differences in inter-site or cross-platform variations. Such differences are part of the gene selection process and have been carried over to the gene ranking/selection stage.

### Evaluation criterion – POG (percentage of overlapping genes)

The POG (percentage of overlapping genes) calculation [11,13] was applied in three types of comparisons: (1) Inter-site comparison using data from the three test sites of each platform; (2) Cross-platform comparison between ABI, AFX, AG1, GEH, and ILM using data from test site 1; for each sample pair, there are ten cross-platform pairs for comparison; (3) Microarray versus TaqMan^® ^assay comparisons.

POG is calculated for many different cutoffs that can be considered as arbitrary.

The number of genes considered as differentially expressed is denoted as 2L, where L is both the number of genes up- and down-regulated. The number of genes available for ranking and selection in one direction, L, varies from 1 to 6000 (with a step of one) or when there are no more genes in one regulation direction, corresponding to 2L varying from 2 to 12,000. Directionality of gene regulation is considered in POG calculations; genes selected by two sites or platforms but with different regulation directionalities are considered as discordant. Therefore, POG can hardly reach 100% in reality.

The formula for calculating POG is: POG = 100*(DD+UU)/2L, where DD and UU are the number of commonly down- or up-regulated genes, respectively, from the two lists, and L is the number of genes selected from the up- or down-regulation directionality. To overcome the confusion of different numbers for the denominator, in our POG calculations we deliberately selected an equal number of up-regulated and down-regulated genes, L [11]. The POG graphs shown in this study are essentially the same as the CAT (correspondence at the top) plots introduced by Irizarry *et al*. [55] and the POG graphs that we introduced previously [11] except that in the current POG graphs the x-axis is in log-scale to emphasize the details when fewer genes are selected.

### Noise-filtering

Most of the analyses in this study exclude flagging information; that is, the entire set of "12,091" genes is used in the analyses. Some calculations are limited to subsets of genes commonly detectable ("common present") by the two test sites or two platforms under comparison. To be denoted as "commonly present", the gene is detected ("present") in the majority of replicates (*e.g*., three or more when there are five replicates) for each sample in a sample-pair comparison and for each test site or platform.

### Gene selection simulation

A simulation was created to emulate the characteristics of the MAQC dataset. Fifteen thousand simulated genes were created where 5,000 were undifferentiated in expression between simulated biological samples A and B and 10,000 were differentiated but at various levels (exponential distribution for the log FC, where almost 4,000 are differentiated two-fold or higher, similar to a typical platform in the MAQC study, divided equally into up and down regulated genes). To simulate instances of technical or biological replicates, multiplicative noise (error) was added to the signal for each gene for each of five simulated replicates for each sample using an error distribution that would produce a replicate CV similar to that typically seen in the MAQC data set (*i.e*., the mean replicate CV would be roughly 10%). The CV for any given gene was randomly selected from a trimmed exponential distribution. To address a variety of additional error scenarios but preserving the same distribution of fold change, we also considered three additional mean CV values (2%, 35%, and 100%). To understand the impact of gene list size on the stability of the DEG list, list sizes of 10, 25, 100, and 500 genes were examined for each mean CV scenario. Several gene selection rules were compared: FC-ranking only, *P*-ranking only, and shrunken t-statistic ranking. Note: *P*-ranking is equivalent to t-statistic ranking as well as ranking based on FDR that monotonically transforms the *P*-value. In addition, shrunken t-statistic ranking is equivalent to ranking based on the test statistic used by SAM and related methods. In addition, rules based on FC-ranking with a *P *threshold were also compared (for *P *= 0.1, 0.01, 0.001, and 0.0001). Finally, to simulate differences in the variation patterns of analytes between platforms and even between laboratories, covariance between laboratories/platforms of the variance for each gene was included in the simulations. For a given mean CV, 20 or more simulated instances of five replicates of simulated biological samples A and B were created and DEG lists were prepared for each instance that were rank ordered using the methods described above. To determine reproducibility of a given method for a given mean CV under a given gene list size, the rank-ordered gene lists from these 20 instances were pair-wise compared for consistency and reproducibility. The results presented in the graphs are averages from those pair-wise comparisons.

The MAQC actual data is characterized by large magnitudes of differential expression among the vast majority of the 12,091 common genes, with some 4000 exhibiting FC > 2 and hundreds with FC > 10. As such, the data may be atypical of commonplace microarray experiments with biological effects. Consequently, two other simulation data sets were created with far fewer genes with large FC, as might be expected in some actual microarray experiments. Specifically, the Small-Delta data set was created with fewer than 50 genes with FC > 2, and a FC < 1.3 for most differentiated genes, and 10,000 undifferentiated genes. In addition, the variances of the genes were correlated similar to that observed in the MAQC data. The third simulated dataset, termed the Medium-Delta set, had a large number of differentiated genes similar to the MAQC simulated dataset, but with small FC similar to the Small-Delta set. Again, gene variances were correlated similar to that observed in the MAQC data.

## Disclaimer

This document has been reviewed in accordance with United States Food and Drug Administration (FDA) policy and approved for publication. Approval does not signify that the contents necessarily reflect the position or opinions of the FDA nor does mention of trade names or commercial products constitute endorsement or recommendation for use. The findings and conclusions in this report are those of the author(s) and do not necessarily represent the views of the FDA. James C. Willey is a consultant for and has significant financial interest in Gene Express, Inc.

## List of abbreviations used

**A**: The MAQC sample A (Stratagene Universal Human Reference RNA); **ABI**: Applied Biosystems microarray platform; **AFX**: Affymetrix microarray platform; **AG1**: Agilent one-color microarray platform; **B**: The MAQC sample B (Ambion Human Brain Reference RNA); **C**: The MAQC sample C (75%A+25%B mixture); **CV**: Coefficient of variation; **D**: The MAQC sample D (25%A+75%B mixture); **DEG**: Differentially expressed genes; **FC**: Fold change in expression levels; **GEH**: GE Healthcare microarray platform; **ILM**: Illumina microarray platform; **MAQC**: MicroArray Quality Control project; ***P***: The *P*-value calculated from a two-tailed two-sample t-test assuming equal variance; **POG**: Percentage of Overlapping (common) Genes between two lists of differentially expressed genes. It is used as a measure of concordance of microarray results.

## Competing interests

The authors declare that they have no competing interests.

## Authors' contributions

LS conceived of, designed, and coordinated the study. LS, WDJ, RVJ, SCH, and RDW carried out the data analyses. LS drafted the manuscript. All authors contributed to the design of the study, the preparation of the manuscript, and the sometimes-heated discussions on the topic of this paper. All authors read and approved the final manuscript.
